# RPflex: A Coarse-Grained Network Model for RNA Pocket Flexibility Study

**DOI:** 10.3390/ijms24065497

**Published:** 2023-03-13

**Authors:** Chen Zhuo, Chengwei Zeng, Rui Yang, Haoquan Liu, Yunjie Zhao

**Affiliations:** Institute of Biophysics and Department of Physics, Central China Normal University, Wuhan 430079, China

**Keywords:** RNA pocket flexibility, flexibility mechanism, interaction characteristics

## Abstract

RNA regulates various biological processes, such as gene regulation, RNA splicing, and intracellular signal transduction. RNA’s conformational dynamics play crucial roles in performing its diverse functions. Thus, it is essential to explore the flexibility characteristics of RNA, especially pocket flexibility. Here, we propose a computational approach, RPflex, to analyze pocket flexibility using the coarse-grained network model. We first clustered 3154 pockets into 297 groups by similarity calculation based on the coarse-grained lattice model. Then, we introduced the flexibility score to quantify the flexibility by global pocket features. The results show strong correlations between the flexibility scores and root-mean-square fluctuation (RMSF) values, with Pearson correlation coefficients of 0.60, 0.76, and 0.53 in Testing Sets I–III. Considering both flexibility score and network calculations, the Pearson correlation coefficient was increased to 0.71 in flexible pockets on Testing Set IV. The network calculations reveal that the long-range interaction changes contributed most to flexibility. In addition, the hydrogen bonds in the base–base interactions greatly stabilize the RNA structure, while backbone interactions determine RNA folding. The computational analysis of pocket flexibility could facilitate RNA engineering for biological or medical applications.

## 1. Introduction

RNA plays major roles in various biological processes, including virus replication, gene transcription, and protein synthesis [1,2,3,4,5]. For example, nucleoside analog inhibitors affect coronavirus disease (COVID-19) replication by binding to virus-dependent RNA polymerase [6]. RNAs are highly dynamic when interacting with other molecules [7,8,9,10]. For example, the L28 protein induces let-7 microRNA to form a specific conformation and inhibits its maturation [11]. The benzimidazole inhibitors of the HCV replicon act by conformational induction of a widened interhelical angle in the IRES subdomain IIa to repress the translation [12]. Thus, the flexibility of RNA structure is closely related to its biological function.

There are some experimental methods to determine the RNA structure. Unfortunately, X-ray crystallography only determines static structures, and RNA structures’ flexibility often prevents the formation of RNA crystals [13]. Although NMR experiments provide multiple dynamic structural models of RNA, the application of solution NMR to RNA is impacted by molecular size [14,15]. Moreover, the computational methods of molecular docking do not consider or implicitly consider conformational changes in RNA [16]. For example, NPDOCKT treats RNA as a rigid body for docking [17], RLDOCK currently only considers the flexibility of ligands in RNA–ligand structures [18], and RNP-devono allows for subtle conformational changes in RNA by folding RNA using Rosetta [19]. Therefore, it is still challenging to improve the accuracy of docking methods due to the limited understanding of the RNA molecules’ flexibility, especially the conformational flexibility of RNA pockets.

At present, many algorithms have been developed for predicting and identifying RNA pockets. For example, 3 V, MSPocket, PocketFinder, and CHUNNEL are used to identify static pockets given a single RNA conformation [20,21,22,23]. Trj_cavity, CAVER 3.0, MDpocket, and Epock track the geometric evolution of pockets throughout the course of molecular dynamics (MD) trajectories [24,25,26,27]. However, these algorithms rarely provide quantitative and detailed analysis of pocket flexibility, and the MD simulations used are computationally quite expensive and time-consuming. While root-mean-square fluctuation (RMSF) can measure pocket flexibility at the atomic level, it ignores the variation in pocket topological features, such as volume, surface area, and sphericity. Previously, we developed the RNA–ligand pocket and RNA–protein pocket databases [28,29]. We analyzed the static features (sequence, secondary structure, and geometry) from the crystal structures or the first NMR structural models in these databases. However, RNAs are relatively dynamic when interacting with ligands or proteins to form complexes. Therefore, there is an urgent need for an approach to quantitatively compute pocket flexibility.

In this work, we provide a computational approach, RPflex, to calculate the conformational flexibility of RNA pockets. The 3154 pockets from 160 NMR RNA-related structures are first divided into 297 groups by similarity using a coarse-grained lattice model. Then, we introduce the flexibility score to quantify the pockets’ topological flexibility. The flexibility scores show a strong correlation with RMSF calculations (Pearson correlation coefficient of 0.53–0.76 in Testing Sets I–IV). The network calculations provide a mechanism for pocket flexibility by network interaction changes. We further analyzed 178 ligand- and 284 protein-binding pockets to reveal the recognition mechanism and interaction characteristics. Our results suggest that most ligand-binding pockets are relatively rigid, and some protein-binding pockets are relatively flexible. We believe RPflex could help the understanding of pocket flexibility to accelerate RNA-related drug design.

## 2. Results

### 2.1. Overview of the RNA Pocket Dataset

**Figure 1:**
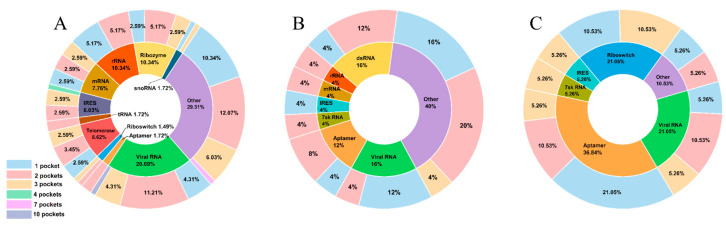
The proportion of various types of RNA and the pocket distribution of each RNA type in RNA (**A**), RNA–ligand (**B**), and RNA–protein (**C**) structures. The inner loop is the proportion of various types of RNA. The outer loop is the maximum number of pockets contained in structural models of a complex.

We divided 160 RNA-related structures of the RNA dataset into thirteen categories based on their functions: rRNA (13), mRNA (10), tRNA (2), viral RNA (32), telomerase RNA (10), snoRNA (2), 7sk RNA (2), dsRNA (4), IRES (9), ribozyme (12), riboswitch (6), aptamer (12), and others (46) (Supplementary Data S1). Figure 1 shows most RNA-related structures (~98%) have no more than three pockets per structural model. To consider pocket flexibility, we also classified the RNAs into four categories based on the number of NMR structural models. Table 1 shows 89% of the RNA-related complexes have no more than 20 structural models. Next, we extracted 3154 pockets from the RNA dataset to form the pocket dataset. Table 1 shows the pocket dataset extracted from RNA (2276 pockets), RNA–ligand (352 pockets), and RNA–protein (526 pockets) complexes. Unlike most datasets that only contain sequence and structure information [30,31,32,33,34], our pocket dataset provides much more detailed information with topological properties: volume, surface area, sphericity, effective radius, and center of mass (Supplementary Data S2). Notably, the pocket dataset provides the flexibility information on the classification of 3154 pockets into 297 pocket groups based on similarity, enabling biologists to better grasp the flexibility of RNA pockets to facilitate drug design or RNA engineering.

### 2.2. RMSF Analysis of Pockets

**Figure 2:**
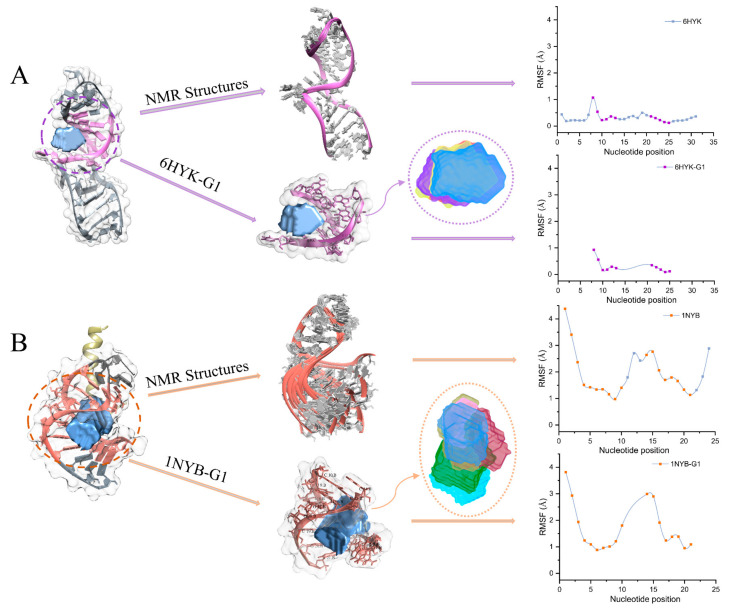
Examples of pocket flexibility reflect structural flexibility. (**A**) Conformational changes and calculations of RMSF for structure (PDB code: 6HYK) and pockets (6HYK-G1). The purple marker dots indicate the overlapping nucleotides forming the pockets in 6HYK-G1. (**B**) Conformational changes and calculations of RMSF for structure (PDB code: 1NYB) and pockets (1NYB-G1). The orange marker dots indicate the overlapping nucleotides forming the pockets in 1NYB-G1.

To compare the pocket flexibility and structural flexibility, we calculated the RMSFs of the pockets and the corresponding structural models. Larger RMSF values indicate higher flexibility, while smaller RMSF values indicate more rigidity. For example, the snoRNA (PDB code: 6HYK) is the U14 snoRNA K-turn motif (kt-U14) determined by NMR. Chagot et al. demonstrated that the structure of kt-U14 is stabilized upon Snu13p binding to control the assembly of many cellular RNPs and their downstream processes [35]. Here, we calculated the RMSFs of 31 nucleotides in 10 NMR structural models and the RMSFs of 11 overlapping nucleotides in a pocket group (6HYK-G1). Figure 2A shows that RMSF values (0.1~1.1 Å) of NMR structures have a similar range to 6HYK-G1 (0.1~0.9 Å). It indicates that the structures and pockets are rigid, as shown by their stable conformational changes. We also observed a similar RMSF trend and an identical nucleotide (U8) with peak RMSF in the NMR structures as in 6HYK-G1. Thus, the rigid NMR structures of 6HYK strongly agree with the pockets’ conformational changes.

Another example is the structure (PDB code: 1NYB) consisting of an amino-terminal bacteriophage φ21 N protein in complex with a boxB RNA. Cilley et al. discovered the φ21 boxB RNA adopts a stem-loop structure with a lack of stable hydrogen bonds [36]. Here, we calculated the RMSFs of 24 nucleotides in 11 NMR structural models and the RMSFs of 18 overlapping nucleotides in a pocket group (1NYB-G1). Figure 2B shows that RMSF values (1.0~4.4 Å) of NMR structures have a similar range to 1NYB-G1 (0.9~3.8 Å). Some nucleotides in NMR structures (~37%) and 1NYB-G1 (~33%) were greater than 2.0 Å. It indicates that the structures and pockets are flexible, as shown by their unstable conformational changes. We also observed a similar RMSF trend and identical nucleotides (G1, G2, and C15) with larger RMSFs in the NMR structures as in 1NYB-G1. Thus, the conformational changes in the flexible NMR structures and pockets of 1NYB are highly consistent.

As mentioned above, the conformational changes in pockets can reflect structural flexibility. Although RMSF can describe pocket flexibility at the atomic level, it ignores the topological features that are essential for pocket-like geometric structures. Therefore, there is an urgent need for a theoretical approach to measuring pocket flexibility in terms of topological characteristics.

### 2.3. Quantitative Analysis of the Pocket Flexibility

Through RPflex, we calculated the flexibility score (Q) to quantify pocket flexibility by the coarse-grained lattice model. Compared to measuring flexibility by RMSF, the flexibility score considers the topological features of pockets, including volume, surface area, and sphericity. A larger value of Q indicates higher flexibility. Then, we divided the pocket dataset into three classes based on the flexibility score: rigidity, 130 pocket groups (0 < Q < 0.30); intermediate flexibility, 119 pocket groups (0.30 ≤ Q < 0.60); and flexibility, 48 pocket groups (0.60 ≤ Q) (Table 2).

**Figure 3:**
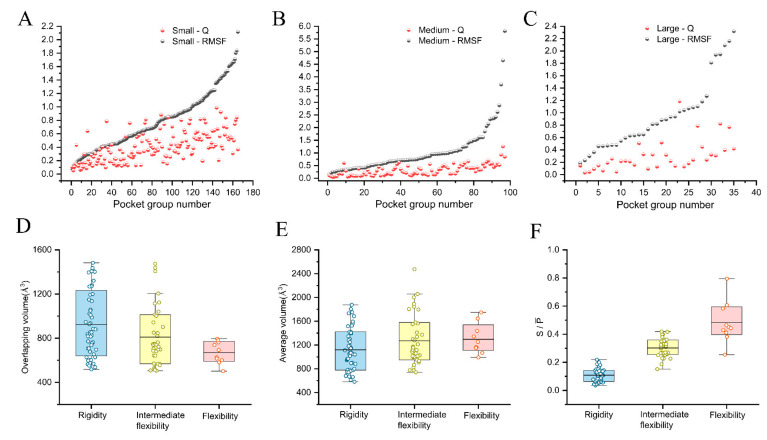
The correlation between the flexibility scores (Qs) and RMSFs of pocket groups in Testing Sets I–III: small (**A**), medium (**B**), and large (**C**). The topological information distribution of overlapping volume (**D**) and average volume (**E**) of pocket groups in the medium. The distribution of $S/\bar{P}$ of pocket groups in the medium (**F**). The mean values are colored black.

To validate the correlation between RMSFs and Qs, we divided the 297 pocket groups into Testing Sets I–III based on the overlapping volume: small ($0<{\;V}_{g}\;\leq \;500\;{Å}^{3}$, 165 pocket groups); medium ($500<V_{g}\;\leq \;1500\;{Å}^{3}$, 97 pocket groups); and large ($1500<{\;V}_{g}\;\leq \;7200\;{Å}^{3}$, 35 pocket groups) (Supplementary Data S3). In Table 2, the binding pockets in small (31.6%) and medium (57.9%) are more than in large (10.5%). It indicates that pockets are mainly distributed in small and medium, including the binding pockets. Similar to that reported by the RPocket database, the volume of the ligand-binding pockets of RNA (~75%) ranged from 200 to 2000 Å^3^ [28]. Next, we further calculated Pearson correlation coefficients to test the pocket flexibility score (Qs) with RMSFs. The results indicate that there are strong correlations for small (Pearson correlation coefficient r = 0.60) and medium (r = 0.76), followed by large (r = 0.53). Figure 3A–C show a similar trend between Qs and RMSFs for pockets in small and medium, followed by large. Therefore, the flexibility scores reflect the conformational changes in pockets, demonstrating the accuracy of RPflex.

Additionally, we tested the flexibility scores by systematically analyzing the topological properties of pockets in the medium. Firstly, we calculated each pocket group’s average volume and overlapping volume. When the average volume is larger, the pocket with a smaller overlapping volume is more flexible. As shown in Figure 3D,E, the average volume tends to decrease in flexibility (1296 ${Å}^{3}$), intermediate flexibility (1271 ${Å}^{3}$), and rigidity (1120 ${Å}^{3}$) pockets, whereas the overlapping volume tends to increase in flexibility (670 ${Å}^{3}$), intermediate flexibility (810 ${Å}^{3}$), and rigidity (923 ${Å}^{3}$) pockets. Secondly, we also calculated the average surface area and overlapping surface area of each pocket group. When the average surface area is larger, the pocket with a smaller overlapping surface area is more flexible. We observed that the average and overlapping surface areas had the same trends as their volumes (Figure S1). Thirdly, we calculated the values of $S/\bar{P}$ for each pocket group. $S/\bar{P}$ reflects the overall spatial variation in pockets in a group, where pockets with larger $S/\bar{P}$ are more flexible. Figure 3F shows the value of $S/\bar{P}$ of flexibility pockets (0.48) is the largest, followed by the intermediate flexibility (0.30) and rigidity (0.11) pockets. Therefore, we verified the accuracy of the flexible score in terms of the topological features.

### 2.4. Flexibility on Binding and Unbinding Pockets

**Figure 4:**
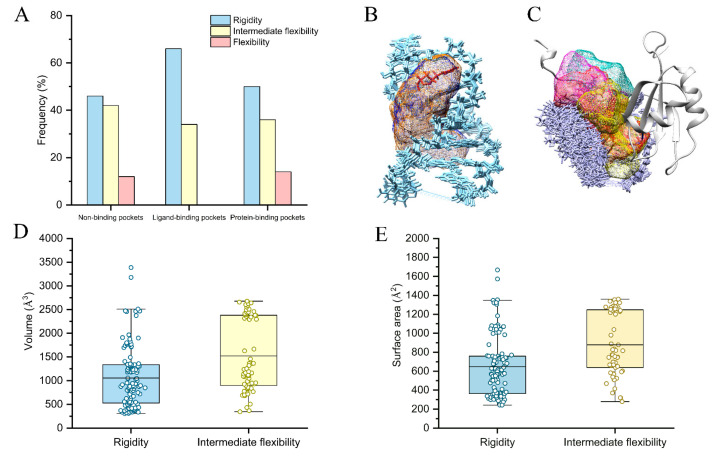
(**A**) The frequency of non-, ligand-, and protein-binding pockets in three classes of flexibility. (**B**,**C**) The examples of structure views of rigidity ligand-binding (2KX8-G2) and flexibility protein-binding pockets (2N82-G1). The topological information distribution of volume (**D**) and surface area (**E**) for ligand-binding pockets. The mean values are colored black.

The primary challenge for RNA-based therapeutics is to determine the targeted pockets. Thus, we explored the flexibility-based recognition mechanism of RNA pockets in our pocket dataset. Figure 4A shows the distribution of non-, ligand-, and protein-binding pockets in three classes: rigidity, intermediate flexibility, and flexibility. The results show that ligands prefer to bind to the rigidity (65.7%) compared to intermediate flexibility (34.3%) pockets, while proteins tend to bind not only primarily to rigidity (50.4%) pockets but also partially to flexibility pockets (14.1%). This may be due to many ligands being small and stable molecules that bind to pockets in a “lock and key” mode. RNA binding proteins (RBP) with disordered regions have higher specificity and affinity toward RNA, and flexible RNAs can induce the conformational transition to their partner RBPs [37,38]. Thus, some protein-binding pockets need to be relatively flexible. We also observed the non-binding pockets, like binding pockets, are mainly distributed in the rigidity class (46.1%), intermediate flexibility class (41.4%), and partially in the flexibility class (12.5%), suggesting their potential as targets for ligands and proteins. The structure views show that conformations of rigidity pockets (2KX8-G2: Q = 0.09) from the RNA–ligand NMR structure (PDB code: 2KX8) are relatively stable with regular nucleotides (Figure 4B), whereas conformations of flexibility pockets (2N82-G1: Q = 0.84) from the RNA–protein NMR structure (PDB code: 2N82) are relatively unstable with disorder nucleotides (Figure 4C).

We further statistically analyzed 178 ligand- and 284 protein-binding pockets to obtain topological principles for the recognition mechanism. Figure 4D,E indicate that the ligand-binding pockets (volume of 1521 ${Å}^{3}$; surface area of 878 ${Å}^{2}$) of intermediate flexibility were more extended than the rigidity pockets (volume of 1055 ${Å}^{3}$; surface area of 647 ${Å}^{3}$). In addition, the rigidity pockets with a volume of 527~1338$\;{Å}^{3}$ and a surface area of 364~759 ${Å}^{2}$ are more likely to bind to small molecules, while the intermediate flexibility pockets with a volume of 899~2381 ${Å}^{3}$ and a surface area of 638~1248 ${Å}^{2}$ (Figure S2). These results emphasize smaller ligand-binding pockets are more rigid compared to larger ones. Moreover, the result implies a trend for protein-binding pockets to be the largest in the rigidity class, followed by the intermediate flexibility and flexibility class (Figure S3).

### 2.5. Physics-Based Interactions on Flexibility

We used the HBPLUS program to calculate the hydrogen bonds and van der Waals contacts of nucleotides that form ligand-binding pockets to understand the flexible mechanisms of RNA. Figure S4A,B show the distributions of the hydrogen bonds and vdW contacts in base–base, base–ribose, base–phosphate, ribose–phosphate, and ribose–ribose, respectively. The distribution details of each pair for both types of contacts are listed in Table S1. It shows more base–base hydrogen bonds (68.0%) in rigidity pockets than in intermediate flexibility pockets (42.2%). Additionally, there are fewer ribose–ribose and ribose–phosphate hydrogen bonds in rigidity pockets (24.6% and 1.5%) than in intermediate flexibility pockets (52.3% and 2.8%). These results suggest that the base–base hydrogen bonds greatly stabilize the RNA structure while hydrogen bonds in backbones determine RNA folding. The vdW contact calculations show that the RNA prefers to form ribose–ribose interactions to optimize the structure in both rigidity (93.7%) and intermediate flexibility (66.7%) pockets. Ligands prefer to form hydrogen bonds and vdW contacts with bases in both rigidity (67.4% and 78.4%) and intermediate flexibility (54.0% and 52.2%) pockets (Figure S4C,D and Table S2). We also observed that both types of contact in ligand–backbone for intermediate flexibility pockets are more than rigidity pockets.

**Figure 5:**
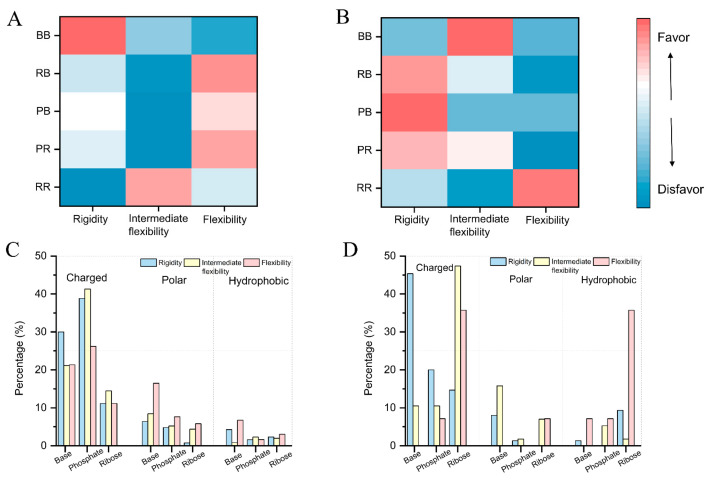
The distribution of hydrogen bonds (**A**) and vdW contacts (**B**) of nucleotides forming protein-binding pockets. The hydrogen bonds (**C**) and vdW contacts (**D**) distribution of proteins with pockets. There are five pairs of interactions: base–base (BB), ribose–base (RB), phosphate–base (PB), phosphate–ribose (PR), and ribose–ribose (RR).

Next, we also calculated hydrogen bonds and van der Waals contacts of nucleotides that form protein-binding pockets (Figure 5A,B and Table S3). The result also shows more base–base hydrogen bonds (58.5%) in rigidity pockets than in flexibility pockets (43.7%). Additionally, the ribose–ribose and ribose–phosphate hydrogen bonds in rigidity pockets (26.1% and 5.7%) are less than in flexibility pockets (37.8% and 6.7%). Similar to ligand-binding pockets, these results also suggest that the hydrogen bonds in base–base interactions considerably limit the flexibility of RNA pockets. According to the vdW contact calculations, RNAs like to generate ribose–ribose connections to optimize the structure. We further analyzed the hydrogen bonds and vdW contacts in pockets with three amino acid categories: charged, polar, and hydrophobic (Figure 5C,D and Table S4). The result shows that the charged amino acids have the highest probability of forming both types of contact with pockets. Additionally, there are more hydrogen bonds in polar and hydrophobic amino acids with flexibility pockets than rigidity pockets.

## 3. Discussion

RNAs interact with other molecules via binding pockets to perform their biological functions [39,40,41,42]. Some of the pockets are flexible and have conformational changes during the binding [43,44,45]. How to quantify pocket flexibility remains one unsolved problem. This work analyzed a large scale of 3154 pockets from 160 non-redundant NMR RNA-related structures. Using the coarse-grained lattice model, the flexibility score (Q) quantifies the flexibility globally based on pockets’ spatial locations and topological properties. The Pearson correlation coefficients between Qs and RMSFs in Testing Sets I–III are 0.60, 0.76, and 0.53, respectively. Therefore, the flexibility score provides a good measure of flexibility through global pocket features. In addition, we analyzed the secondary structures for 30 rigidity and 30 flexibility pocket groups. The results show that the stem units are relatively more rigid than the loop units (Supplementary Data S4). 

**Figure 6:**
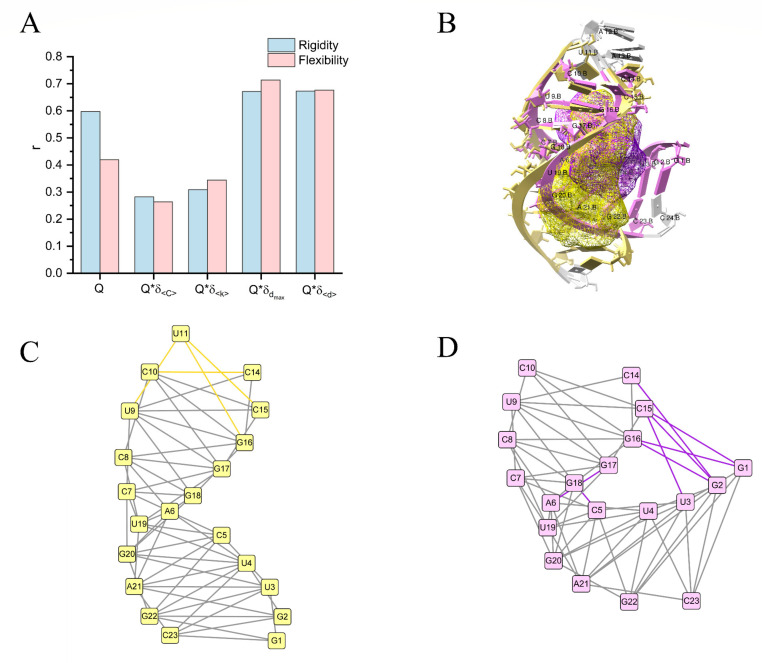
(**A**) The Pearson correlation coefficients (r) between RMSF and Q, ${Q*\sigma }_{<C>}$, ${Q*\sigma }_{<k>}$, ${Q*\sigma }_{d_{\max}}$, and ${Q*\sigma }_{<d>}$, respectively. (**B**) The nucleotides forming the yellow pocket in NMR structural model 1 (PDB code: 1NYB) are colored yellow, and those forming the purple pocket in NMR structural model 10 (PDB code: 1NYB) are colored purple. The nucleotides that do not form the pockets are colored gray. (**C**,**D**) The pocket networks of NMR structural model 1 (PDB code: 1NYB, colored yellow) and NMR structural model 10 (PDB code: 1NYB, colored purple). The overlapping edges between NMR structural model 1 and NMR structural model 10 are colored gray, and the different edges are colored yellow and purple, respectively.

Since the coarse-grained lattice model considers neighboring interatomic interactions, we propose the network model to capture the effects of interactions between nucleotides on RNA folding and, subsequently, on pocket flexibility. The pocket network is defined by nucleotides as nodes and non-covalent interactions as edges. Here, we calculated the standard deviation of average degrees ($\sigma_{<k>}$) and the standard deviation of average clustering coefficients ($\sigma_{<C>}$) to measure the local interaction changes, while the standard deviation of diameters ($\sigma_{d_{max}}$) and the standard deviation of average path lengths ($\sigma_{<d>}$) to measure both local and long-range interaction changes (Supplementary Data S5). Combining the flexibility score (Q), the results show that the local interaction calculations (${Q*\sigma }_{<k>}$ and ${Q*\sigma }_{<C>}$) decrease the Pearson correlation coefficients to 0.34 and 0.26 in flexible pockets, respectively (Figure 6A). In contrast, the Pearson correlation coefficients increase to 0.71 and 0.68 when considering both local and long-range interactions (${Q*\sigma }_{d_{max}}$ and ${Q*\sigma }_{<d>}$). It indicates that the long-range interaction changes contributed most to flexibility. Therefore, the network calculations provide a flexibility mechanism by network interaction changes and better characterizes pocket flexibility by integrating flexibility score (Q).

The network model provides both local and long-range interaction information. For example, the structure (PDB code: 1NYB) consists of an amino-terminal bacteriophage φ21 N protein in complex with a boxB RNA [36]. Comparing the NMR structural model 1 (PDB code: 1NYB), the nucleotides C5, A6, G17, and G18 form two more interactions in the NMR structural model 10 (PDB code: 1NYB), leading to structural bending during RNA folding (Figure 6B–D). The shortest path communications are G1–U3–C5–C7–U9–C15 and G2–U4–A6–C8–G16–C14 in the NMR structural model 1. However, the long-range interaction communications changed to G1–C15 and G2–C14 direct interactions by structural bending in the NMR structural model 10. Thus, long-range interactions play an essential role in the flexibility of RNA pockets.

The structural elements have been successfully applied to RNA complex structure prediction. However, understanding the higher level of pocket flexibility is still limited. To test if the higher level of pocket flexibility can identify native-like RNA complex structures, we ran the popular RNA structure prediction program (RLDOCK) on complex structures (PDB code: 1ARJ and 1F7I) to build sampling structures and evaluate the prediction accuracy. For topological principles of the recognition mechanism, the rigidity pockets with a volume of 527~1338 ${Å}^{3}$ and a surface area of 364~759 ${Å}^{2}$ are more likely to bind to small molecules, while the intermediate flexibility pockets with a volume of 899~2381 ${Å}^{3}$ and a surface area of 638~1248 ${Å}^{2}$. Thus, we divided the prediction structures into global sampling, pocket sampling, and target pocket sampling classes (Table S5). Figure S5 shows the all-atom root-mean-square deviation (RMSD) measured against the native structure. For 1ARJ and 1F7I, the top 20 predictions for target pocket sampling show lower RMSDs (5.95 ± 2.43 Å and 7.85 ± 0.56 Å) than global sampling (14.63 ± 1.49 Å and 9.04 ± 1.84 Å) and pocket sampling (14.34 ± 1.27 Å and 8.33 ± 0.91 Å). The results suggest that the higher level of pocket flexibility patterns may improve the RNA complex structure prediction.

## 4. Materials and Methods

### 4.1. Structure Dataset Collection

To construct a diverse RNA dataset, we extracted the RNA-related structures from the Protein Data Bank (20 October 2021) with the search options “RNA” and “NMR”. We extracted the RNA-related structures with a single RNA chain that binds to ligands or a single-stranded protein. There are three types of NMR structures: RNA (358 entries), RNA–ligand (54 entries), and RNA–protein (41 entries). Then, we removed the short (less than 20 nucleotides) and highly complex (more than 120 nucleotides) RNA structures. To acquire the non-redundant dataset, we used the CD-hit to remove the redundant RNAs with RNA sequence identities >80% [46,47]. Then, the dataset consisted of 143 RNA structures, 24 RNA–ligand structures, and 27 RNA–protein structures. In addition, the NMR structures with one structural model were removed. We also removed complexes if there were less than three pockets in a group (see the method Section 2.2). Thus, the RNA dataset includes 116 RNA structures, 19 RNA–ligand structures, and 25 RNA–protein structures.

To construct the pocket dataset, the pockets of an NMR structure with multiple structural models were recognized by the 3V server using the rolling probe method [20,48,49]. The coordinates of the structural model are superimposed on the cubic grids to roll two virtual probes on the molecular surface. If the probe contacts more than two atoms on the molecule surface, then the center of the probe is recorded [48]. These discrete positions form the boundary of the pocket [49]. Here, we detected pockets using the discrete volume method, where the default large probe radius is 10 Å, and the small probe radius is 3 Å. The effective radius ($r_{\mathrm{eff}}$) and sphericity (ψ) were calculated by

(1) $$r_{\mathrm{eff}}=\frac{3V_{P}}{A_{P}},\;\psi =\frac{\pi^{1/3}{\left(6V_{p}\right)}^{2/3}}{A_{P}}$$

where $V_{p}$ and $A_{p}$ represent the volume and surface area of the pocket, respectively.

### 4.2. Criteria for Pocket Conformational Flexibility

**Figure 7:**
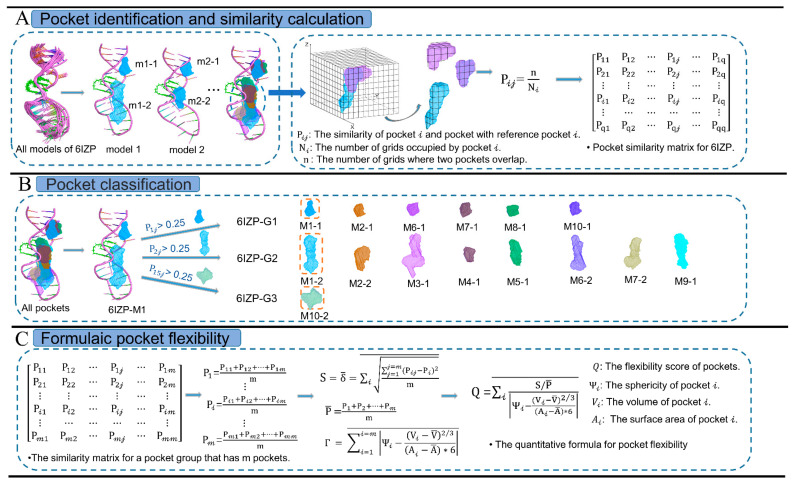
The workflow of the quantifying pockets’ flexibility. (**A**) Calculating the similarity of pockets of all the structural models of a complex, such as an RNA–ligand complex (PDB code: 6IZP). (**B**) Dividing pockets into different groups based on pocket similarity. (**C**) Calculating the flexibility score of a pocket group.

Here, we focus on exploring the conformational flexibility of RNA pockets, using the flexibility score to quantitatively describe topological changes globally. The flexibility score is calculated by the geometric topology of pockets using the coarse-grained lattice model. The coarse-grained lattice model scales the box for the pocket size and divides the box space into 3D grids of small cubes (i.e., voxels). Figure 7 shows the workflow using one RNA–ligand structure (PDB code: 6IZP) as an example.

The first step is to calculate the pocket similarity using the coarse-grained lattice model (Figure 7A). For q pockets from all structural models of an NMR structure, our method generated a pocket similarity matrix (q × q) describing the similarity of any two pockets. To describe the index of the matrix element, we labeled the pocket numbers in the order of the structural models as 1, 2, 3 …, q. For example, all the pockets of 6IZP were labeled as 1 (m1-1), 2 (m1-2), 3 (m2-1), …, 15 (m10-2). In our model, the coordinates of two pocket conformations are superimposed on the cubic grids in the box. The box size is scaled according to the two pockets stored at a time, ensuring full use of the box space to save computing time. The box is divided into voxels of size $1\;{Å}^{3}$, and the value of the voxel occupied by the pocket is 1; otherwise, it is 0. The overlapping voxel value of the two pockets is 2. Here, the matrix element, $P_{𝒾𝒿}$, is the similarity between pocket 𝒾 and pocket 𝒿, where pocket 𝒾 is selected as the reference conformation. We count the number of voxels ($N_{𝒾}$) occupied by pocket 𝒾 and the number of overlapping voxels ($n_{𝒾𝒿}$). Thus, pocket similarity, $P_{𝒾𝒿}$, describes the conformational changes between two pockets in spatial location, calculated by

(2) $$P_{𝒾𝒿}=\frac{n_{𝒾𝒿}}{N_{𝒾}}$$

Then, we figured out $P_{𝒾𝒿}$ between any two of q pockets to generate a pocket similarity matrix, where $P_{𝒾𝒾}=1$.

The second step is to divide q pockets into different pocket groups based on pocket similarity (Figure 7B). Here, we chose $P_{𝒾𝒿}=0.25$ as the cutoff for pocket classification, which means that pocket 𝒾 and pocket 𝒿 are treated as a group for $P_{𝒾𝒿}>0.25$. We used the pocket positions on the first structural model as the reference conformations, such as two pockets in the first structural model of 6IZP. For the pocket similarity matrix of 6IZP, six pockets were picked to form 6IZP-G1 for $P_{1𝒿}>0.25$, and eight pockets were picked to form 6IZP-G2 for $P_{2𝒿}>0.25$. Then only one pocket left was counted as 6IZP-G3. The research objects were defined as pocket groups with no less than three pockets; otherwise, the pocket groups were deemed invalid, such as 6IZP-G3. Through this step, we can divide the pockets obtained from the NMR structure into different groups according to their spatial position and then obtain the pocket similarity matrixes of different groups to calculate the pocket flexibility.

The third step is to calculate the flexibility score to quantify the conformational flexibility of each pocket group (Figure 7C). For 𝓂 pockets in a group, the flexibility score describes pocket conformational changes in two ways: spatial location and topology properties. For the spatial location, we calculated the standard deviation of similarity for pocket 𝒾, $\sigma_{𝒾}$, which reflects the spatial variation in 𝓂 pockets with reference pocket 𝒾. Considering the general trend of conformational changes in 𝓂 pockets, the average of the standard deviation of similarity (S) and the average of the similarity matrix ($\bar{P}$) were calculated by

(3) $${\;\sigma }_{𝒾}=\sqrt{\frac{\sum_{𝒿=1}^{𝒿=𝓂}{\left(P_{𝒾𝒿}-\bar{\sum_{\ell =1}^{\ell =𝓂}P_{𝒾\ell }}\right)}^{2}}{𝓂}}$$

(4) $$S\;=\frac{\sigma_{1}+\sigma_{2}+\cdot \cdot \cdot +\sigma_{𝓂}}{𝓂}$$

(5) $$\bar{P}=\frac{\sum_{𝒾=1}^{𝒾=𝓂}\sum_{𝒿=1}^{𝒿=𝓂}P_{𝒾𝒿}}{𝓂\times 𝓂}$$

$\bar{P}$ reflects the degree of spatial overlap of the 𝓂 pockets. A larger value of S means more flexibility, whereas a larger $\bar{P}$ value means more rigidity. Thus, the value of $S/\bar{P}$ reflects the overall situation of the spatial variation in 𝓂 pockets. For topology properties, we considered the trend between ($V_{𝒾}-\bar{V})$ and ($A_{𝒾}-\bar{A})$ for pocket 𝒾. $\bar{V}$ and $\bar{A}$ are the average volume and average surface area of 𝓂 pockets, respectively. According to the definition of sphericity, we define Γ to describe the topological changes in 𝓂 pockets, calculated by

(6) $$\Gamma \;=\bar{\sum_{𝒾=1}^{𝒾=𝓂}\left|\Psi_{𝒾}-\frac{{\left(V_{𝒾}-\bar{V}\right)}^{2/3}}{6(A_{𝒾}-\bar{A})}\right|}$$

where $\Psi_{𝒾}$ is the sphericity of pocket 𝒾. Thus, for 𝓂 pockets in a pocket group, the flexibility score, Q, is calculated by

(7) $$Q\;=\frac{S/\bar{P}}{\Gamma }=\bar{\sum_{𝒾=1}^{𝒾=𝓂}\frac{S/\bar{P}}{\left|\Psi_{𝒾}-\frac{{\left(V_{𝒾}-\bar{V}\right)}^{2/3}}{6(A_{𝒾}-\bar{A})}\right|}}$$

Larger Q values indicate higher flexibility, while lower Q values indicate more rigidity.

### 4.3. Network Construction

We constructed structure networks to capture the effect of interactions between nucleotides on pocket flexibility. The fragment of RNA tertiary structure forming a pocket is transformed into a network in which nucleotides are nodes, and non-covalent interactions with each other are edges. Previous work has shown that 8 Å can serve as a reliable contact cutoff for RNA tertiary structure studies [50]. If two discrete nucleotides in a sequence contain a pair of heavy atoms and are less than 8 Å apart, the two nucleotides are connected by an edge. Cytoscape was used for network visualization [51].

To characterize the network characteristics, we calculated the average degree (<k>) and average clustering coefficient (<C>) to infer the local interactions, while the diameter ($d_{\max}$) and average path length (<d>) to infer both local and long-range interactions. We further calculated the standard deviations of four characteristics to measure the flexibility of pocket networks. The formulas are as follows:

The average degree, <k>, indicates the average number of connected edges per node, calculated by

(8) $$<k>=\frac{1}{N}\sum_{𝒾=1}^{N}k_{𝒾}=\frac{2L}{N},\;\sigma_{<k>}=\sqrt{\frac{\sum_{𝒿=1}^{𝒿=𝓂}{\left({<k>}_{𝒿}-\bar{\sum_{\ell =1}^{\ell =𝓂}{<k>}_{\ell }}\right)}^{2}}{𝓂}}$$

where $k_{𝒾}$ denotes the degree of node 𝒾, N is the total number of nodes, L is the total number of connected edges in the network, and $\sigma_{<k>}$ represents the standard deviation of the average degree for 𝓂 pocket networks.

The average clustering coefficient, <C>, denotes the aggregation density of the whole network, defined as

(9) $$<C>=\frac{1}{N}\sum_{𝒾=1}^{N}\frac{2L_{𝒾}}{k_{𝒾}\left(k_{𝒾}-1\right)},\;\sigma_{<C>}=\sqrt{\frac{\sum_{𝒿=1}^{𝒿=𝓂}{\left({<C>}_{𝒿}-\bar{\sum_{\ell =1}^{\ell =𝓂}{<C>}_{\ell }}\right)}^{2}}{𝓂}}$$

where $L_{𝒾}$ is the number of connected edges between the $k_{𝒾}$ neighbors of node 𝒾, and $\sigma_{<C>}$ represents the standard deviation of the average clustering coefficient for 𝓂 pocket networks.

The diameter, $d_{\max}$, is the maximum distance between all pairs of nodes in the network. The standard deviation of the diameter for 𝓂 pocket networks is calculated by

(10) $$\sigma_{d_{max}}=\sqrt{\frac{\sum_{𝒿=1}^{𝒿=𝓂}{\left(d_{\max}{}_{𝒿}-\bar{\sum_{\ell =1}^{\ell =𝓂}d_{\max}{}_{\ell }}\right)}^{2}}{𝓂}}$$

The average path length, <d>, is defined as the average distance between any two nodes, calculated by

(11) $$<d>=\frac{2}{N\left(N-1\right)}\sum_{𝒾\geq ℊ}d_{𝒾ℊ},\;\sigma_{<d>}=\sqrt{\frac{\sum_{𝒿=1}^{𝒿=𝓂}{\left({<d>}_{𝒿}-\bar{\sum_{\ell =1}^{\ell =𝓂}{<d>}_{\ell }}\right)}^{2}}{𝓂}}$$

where $d_{𝒾ℊ}$ is the shortest path between node 𝒾 and node ℊ, and $\sigma_{<d>}$ represents the standard deviation of the average path length for 𝓂 pocket networks.

### 4.4. RMSF Calculation

Root-mean-square fluctuation (RMSF) verified the pocket flexibility by using GROMACS. Here, we used overlapping nucleotides to measure flexibility. We define the overlapping nucleotides as a nucleotide that occurs more than 50% in a pocket group. The overlapping nucleotides forming the first pocket in each pocket group were used as the reference positions. For a pocket group, the nucleotide-averaged RMSF is calculated by

(12) $$\mathrm{RMSF}\;=\frac{\sum_{𝒾}^{\ell }\sqrt{\frac{\sum_{𝒿}^{𝓂}{\left(X_{𝒿}-{\tilde{X}}_{𝒿}\right)}^{2}}{𝓂}}}{\ell }$$

where $\left(X_{𝒿}-{\tilde{X}}_{𝒿}\right)$ is the position of a nucleotide minus its reference positions, 𝓂 is the total number of pockets, and ℓ  is the number of overlapping nucleotides. To compare pocket flexibility and RNA structural flexibility, we calculated RMSF for structural models. The structural model of the first pocket in each pocket group was used as the reference positions.

For a pocket group, $\bar{V}$ is the average volume of pockets, and $\bar{P}$ is the average of the pocket similarity matrix. Then, we specify $V_{g}$ ($V_{g}=\bar{V}\times \bar{P}$) as the overlapping volume of pockets in the group. As mentioned above, the overlapping nucleotides are used for RMSF calculations, and the pocket flexibility score incorporates the $\bar{P}.$ Thus, the overlapping volume, $V_{g}$, is a good feature for building testing sets. To validate the correlation between RMSFs and Qs, we divided pocket groups into Testing Sets I–III based on overlapping volume: small ($0<{\;V}_{g}\;\leq \;500\;{Å}^{3}$), medium ($500<{\;V}_{g}\;\leq \;1500\;{Å}^{3}$), and large ($1500<{\;V}_{g}\;\leq \;7200\;{Å}^{3}$). Next, we tested the network model on Testing Set IV with 30 rigid pocket groups (324 pockets) and 30 flexible pocket groups (257 pockets).

### 4.5. Chemical Group and Interaction Calculation

We utilize the atom-level model for interaction calculation. First, we consider the nucleotides as six chemical groups: phosphate, ribose, adenine, cytosine, guanine, and uracil (Table S6). For protein, 20 amino acid chemical groups are classified into three categories according to their physicochemical properties: charged residues (Asp, Glu, Lys, Arg, and His), polar residues (Cys, Asn, Gln, Ser, Thr, and Tyr), hydrophobic residues (Ala, Phe, Gly, Ile, Leu, Trp, Met, Pro, and Val) (Table S7). Then, we used the HBPLUS program to calculate the pockets’ hydrogen bonds and van der Waals contacts [52]. Here, the criteria for defining a hydrogen bond were hydrogen–acceptor distance <2.7 Å and donor–acceptor distance <3.35 Å. The van der Waals contacts were defined as all contacts between atoms not involved in hydrogen bonds that were <3.9 Å apart. Next, we obtained the secondary structure of RNA from the Forna server [53].

## 5. Conclusions

In this work, we developed the RPflex to analyze the flexibility of RNA pockets. This new approach considers the topological properties and three-dimensional conformation of pockets. RPflex uses a coarse-grained network model to quantify flexibility by pocket features. Combining the network interactions, our method provides local and long-range interaction information and better characterizes pocket flexibility. The result shows that the ligand-binding pockets prefer to be more rigid compared to protein-binding pockets. The base–base hydrogen bonds stabilize the RNA structure, and ribose–ribose van der Waals contacts optimize the structure. In addition, we constructed a pocket dataset that provides information on topology properties and flexibility characteristics. We hope that the analysis of pocket flexibility will facilitate the discovery of druggable pockets and contribute to future drug design.

## Figures and Tables

**Table 1:** Summary of RNA dataset based on the number of structural models.

Structure Type	Number of Models	Complexes	Nucleotides	RNA Pockets
RNA	5~10	47	2149	655
	11~20	56	1894	1260
	21~30	12	405	322
	31~40	1	31	39
	Total	116	4479	2276
RNA–ligand	5~10	12	432	155
	11~20	5	183	119
	21~30	1	38	20
	31~40	1	27	58
	Total	19	680	352
RNA–protein	5~10	6	215	59
	11~20	16	493	332
	21~30	1	27	21
	31~40	2	60	114
	Total	25	795	526

**Table 2:** The distribution of the classes of flexibility and binding types for pocket groups in Testing Sets I–III.

Testing Set	Number of Pocket Groups	Class			Binding Type		
		Rigidity	Intermediate Flexibility	Flexibility	Non-Binding	Ligand-Binding	Protein-Binding
Small	165	58	73	34	153	2	10
Medium	97	50	37	10	75	11	11
Large	35	22	9	4	31	2	2
Total	297	130	119	48	259	15	23

## Data Availability

The RNA dataset, the pocket dataset, and the code guidance are available at http://www.zhaoserver.com.cn/RPflex/RPflex.html, accessed on 16 February 2023.

## Supplementary Materials

The following supporting information can be downloaded at: https://www.mdpi.com/article/10.3390/ijms24065497/s1.
