# Supplementary material for: RPflex: A Coarse-Grained Network Model for RNA Pocket Flexibility Study
Source: Int J Mol Sci. 2023 Mar 13;24(6):5497. doi: 10.3390/ijms24065497 (PMC10058308; doi:10.3390/ijms24065497)
Supplement: Supplementary file 1 [file ijms-24-05497-s001.zip › Supplementary Information.pdf]

International Journal of
Molecular Sciences

MDPI

Supplementary Information

RPflex: A coarse-grained network model for RNA pocket flexibility study

Chen Zhuo, Chengwei Zeng, Rui Yang, Haoquan Liu, and Yunjie Zhao*

Institute of Biophysics and Department of Physics, Central China Normal University, Wuhan, 430079, China

* Correspondence: yjzhaowh@mail.ccnu.edu.cn

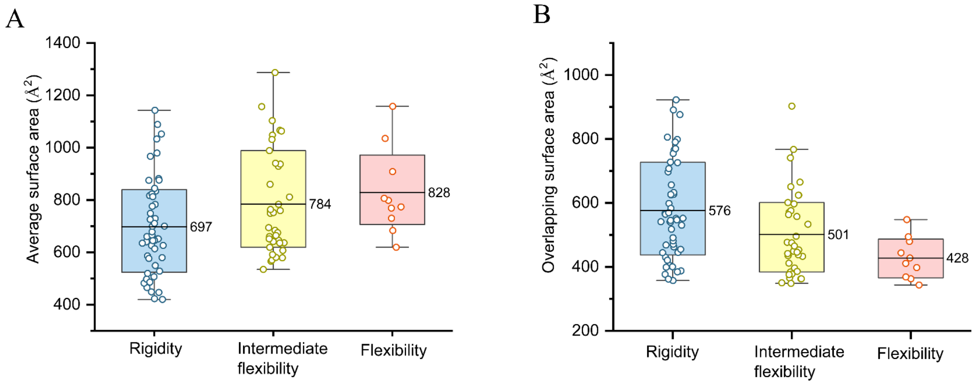

Figure S1. The distribution of average surface area (A) and overlapping surface area (B) of pocket groups in the medium. The mean values are colored black.

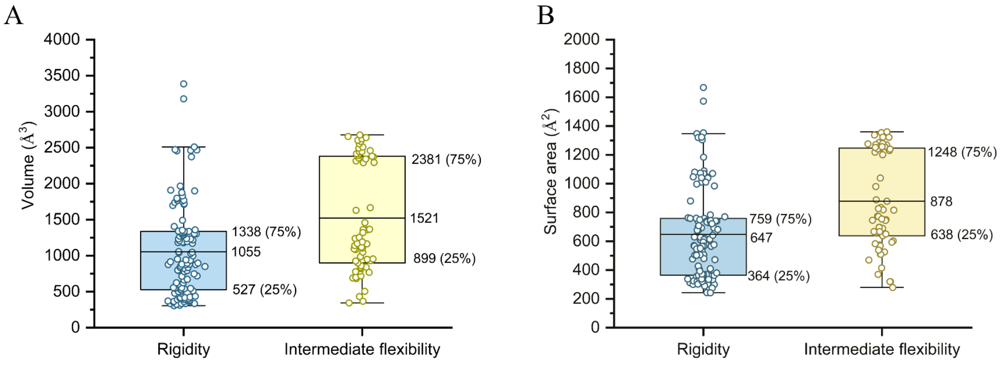

Figure S2. The topological information distribution of volume (A) and surface area (B) for ligand-binding pockets in rigidity and intermediate flexibility. The mean values, ranges of the box, and percentages are marked beside each box.

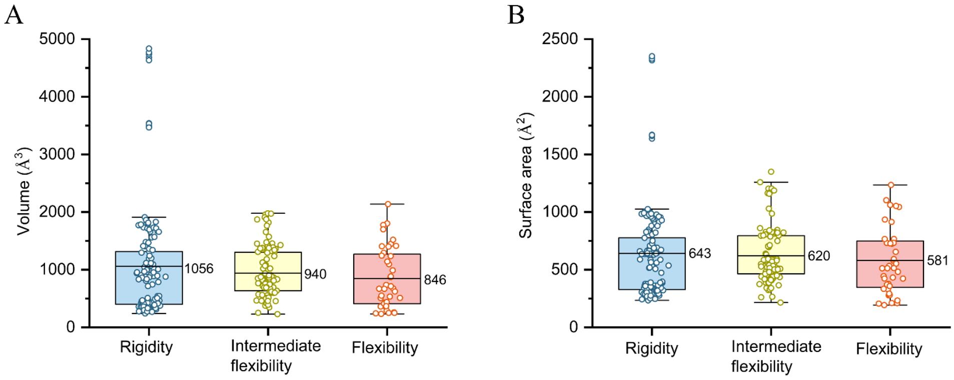

Figure S3. The topological information distribution of volume (A) and surface area (B) for protein-binding pockets in rigidity, intermediate flexibility, and flexibility. The mean values are colored black.

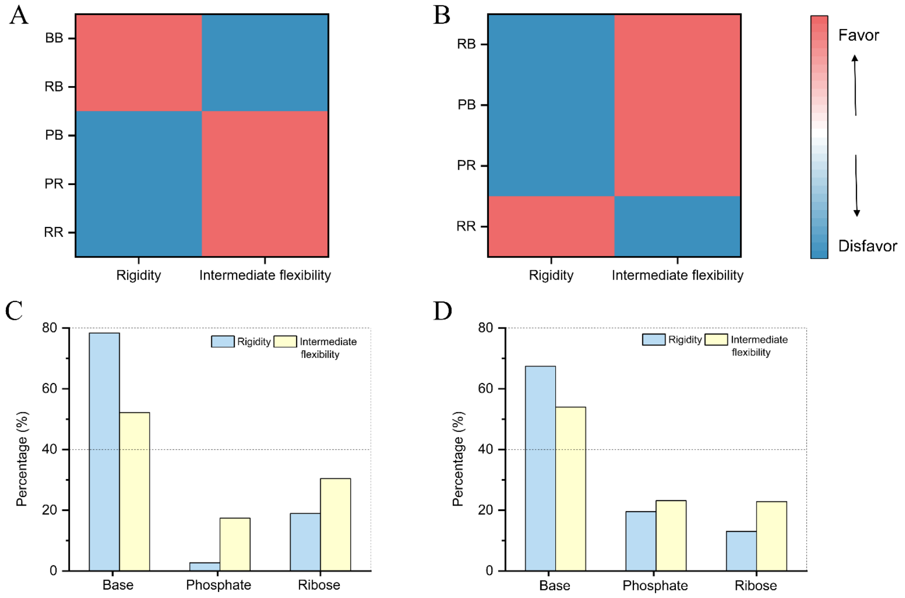

Figure S4. The distribution of hydrogen bonds (A) and vdW contacts (B) of nucleotides forming ligand-binding pockets. The hydrogen bonds (C) and vdW contacts (D) distribution of ligands with pockets. There are five pairs of interactions: base-base (BB), ribose-base (RB), phosphate-base (PB), phosphate-ribose (PR), and ribose-ribose(RR).

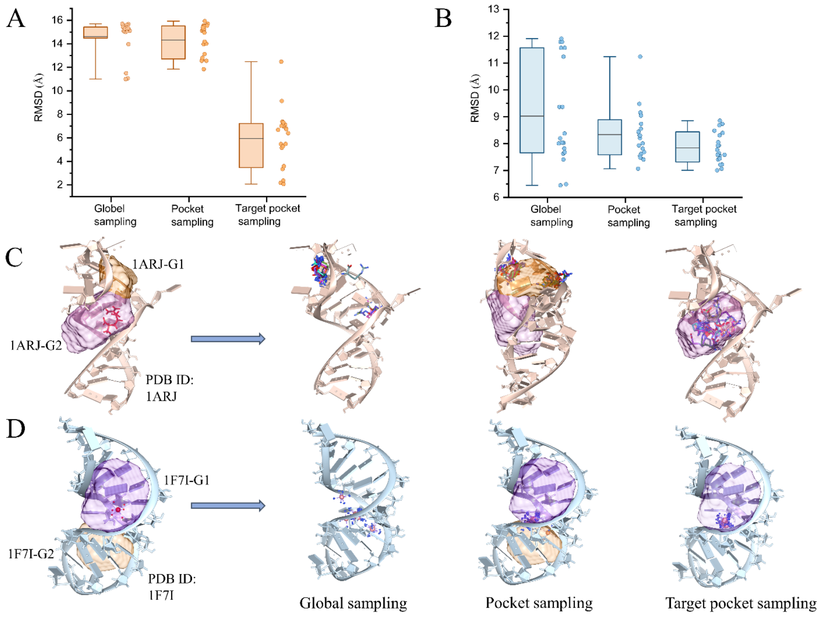

Figure S5. (A) The RMSD values for sampling in the RNA-ligand structure (PDB ID: 1ARJ). (B) The RMSD values for sampling in the RNA-ligand structure (PDB ID: 1F7I). (C, D)The structural views for three sampling types in RNA-ligand structures using the RLDOCK program.

Table S1. The distribution of hydrogen bonds and Van der Waals contacts of nucleotides forming ligand-binding pockets.

Pair [1]	Hydrogen bonds		Van der Waals contacts	
	Rigidity	Intermediate flexibility	Rigidity	Intermediate flexibility
BB	968 (68.0%)	539 (42.2%)	0	0
RB	72 (5.1%)	22 (1.7%)	5 (6.3%)	2 (9.5%)
PB	11 (0.8%)	13 (1.0%)	0	3 (14.3%)
PR	22 (1.5%)	36 (2.8%)	0	2 (9.5%)
RR	350 (24.6%)	668 (52.3%)	74 (93.7%)	14 (66.7%)
Total	1423	1278	79	21

[1] There are five interacting pairs of chemical compositions in ligand-binding pockets: base-base (BB), ribose-base (RB), phosphate-base (PB), phosphate-ribose (PR), and ribose-ribose (RR).

Table S2. The distribution of hydrogen bonds and Van der Waals contacts between ligands and pockets.

Type [1]	Hydrogen bonds		Van der Waals contacts	
	Rigidity	Intermediate flexibility	Rigidity	Intermediate flexibility
Base	569 (67.4%)	170 (54.0%)	58 (78.4%)	12 (52.2%)
Phosphate	165 (19.5%)	73 (23.2%)	2 (2.7%)	4 (17.4%)
Ribose	110 (13.1%)	72 (22.8%)	14 (18.9%)	7 (30.4%)
Total	844	315	74	23

[1] Three chemical groups interact with ligands: base, phosphate, and ribose.

Table S3. The distribution of hydrogen bonds and Van der Waals contacts of nucleotides forming protein-binding pockets.

Pair [1]	Hydrogen bonds			Van der Waals contacts		
	Rigidity	Intermediate flexibility	Flexibility	Rigidity	Intermediate flexibility	Flexibility
BB	1283 (58.5%)	885 (47.5%)	181 (43.7%)	2 (3.7%)	27 (45.8%)	0
RB	173 (7.9%)	94 (5.0%)	41 (9.9%)	6 (11.1%)	4 (6.8%)	0
PB	39 (1.8%)	5 (0.3%)	8 (1.9%)	3 (5.6%)	0	0
PR	124 (5.7%)	59 (3.2%)	28 (6.7%)	11 (20.4%)	10 (16.9%)	0
RR	573 (26.1%)	820 (44.0%)	157 (37.8%)	32 (59.2%)	18 (30.5%)	8 (100%)
Total	2192	1863	415	54	59	8

[1] There are five interacting pairs of chemical compositions in protein-binding pockets: base-base (BB), ribose-base (RB), phosphate-base (PB), phosphate-ribose (PR), and ribose-ribose (RR).

Table S4. The distribution of hydrogen bonds and Van der Waals contacts between proteins and pockets.

Type [1]	Hydrogen bonds			Van der Waals contacts		
	Rigidity	Intermediate flexibility	Flexibility	Rigidity	Intermediate flexibility	Flexibility
(Charged) [2]						
Base	401 (30.0%)	203 (21.1%)	92 (21.3%)	34 (45.3%)	6 (10.5%)	0
Phosphate	519 (38.8%)	397 (41.3%)	113 (26.2%)	15 (20.0%)	6 (10.5%)	1 (7.1%)
Ribose	149 (11.1%)	139 (14.5%)	48 (11.1%)	11 (14.7%)	27 (47.4%)	5 (35.7%)
(Polar) [3]						
Base	85 (6.4%)	81 (8.4%)	71 (16.5%)	6 (8.0%)	9 (15.8%)	0
Phosphate	64 (4.8%)	50 (5.2%)	33 (7.7%)	1 (1.3%)	1 (1.8%)	0
Ribose	10 (0.7%)	42 (4.4%)	25 (5.8%)	0	4 (7.0%)	1 (7.1%)
(Hydrophobic) [4]						
Base	57 (4.3%)	8 (0.8%)	29 (6.7%)	1 (1.3%)	0	1 (7.1%)
Phosphate	21 (1.6%)	22 (2.3%)	7 (1.6%)	0	3 (5.3%)	1 (7.1%)
Ribose	31 (2.3%)	19 (2.0%)	13 (3.0%)	7 (9.3%)	1 (1.8%)	5 (35.7%)
Total	1337	961	431	75	57	14

[1] There are three chemical groups of pockets: base, phosphate, and ribose; [2, 3, 4] Twenty amino acid chemical groups of proteins are classified into three categories: charged, polar, and hydrophobic.

Table S5. The information of pocket groups for the tested RNAs.

PDB ID	Pocket group	Flexibility Score (Q)	Class	Average volume (Å^3)	Average surface area（Å^2）	Target pocket
1ARJ	1ARJ-G1	0.46	Intermediate flexibility	327	281	×

	1ARJ-G2	0.47	Intermediate flexibility	993	666	√
1F7I	1F7I-G1	0.26	Rigidity	530	376	√
	1F7I-G2	0.28	Rigidity	312	259	×

Table S6. The atomic composition of RNA chemical groups.

Chemical group	Atomic composition
Phosphate	P , OP1, OP2
Ribose	C1’, C2’, C3’, C4’, C5’, H1’, H2’, H3’, H4’, H5’, H5’’, O2’, O3’, O4’, O5’, HO2’
Adenine	C2 , C4, C5, C6, C8, H2, H8, H61, H62, N1, N3, N6, N7, N9
Guanine	C2, C4, C5, C6, C8, H1, H8, H21, H22, O6, N1, N2, N3, N7, N9
Cytosine	C2, C4, C5, C6, H5, H6, H41, H42, O2, N1, N3, N4
Uracil	C2, C4, C5, C6, H3, H5, H6, O2, O4, N1, N3

Table S7. The atomic composition of 20 amino acid chemical groups.

Chemical group	Atomic composition
Asp	N, CA, C, O, CB, CG, OD1, OD2, H, HA, HB2, HB3
Glu	N, CA, C, O, CB, CG, CD, OE1, OE2, H, HA, HB2, HB3, HG2, HG3
Lys	N, CA, C, O, CB, CG, CD, CE, NZ, H, HA, HB2, HB3, HG2, HG3, HD2, HD3, HE2, HE3, HZ1, HZ2, HZ3
Arg	N, CA, C, O, CB, CG, CD, NE, CZ, NH1, NH2, H, HA, HB2, HB3, HG2, HG3, HD2, HD3, HE, HH11, HH12, HH21, HH22
His	N, CA, C, O, CB, CG, ND1, CD2, CE1, NE2, H, HA, HB2, HB3, HD1, HD2, HE1, HE2
Cys	N, CA, C, O, CB, SG, H, HA, HB2, HB3, HG
Asn	N, CA, C, O, CB, CG, OD1, ND2, H1, H2, HA, HB2, HB3, HD21, HD22
Gln	N, CA, C, O, CB, CG, CD, OE1, NE2, H, HA, HB2, HB3, HG2, HG3, HE21, HE22

Ser	N, CA, C, O, CB, OG, H1, H2, H3, HA, HB2, HB3, HG
Thr	N, CA, C, O, CB, OG1, CG2, H1, H2, HA, HB, HG1, HG21, HG22, HG23
Tyr	N, CA, C, O, CB, CG, CD1, CD2, CE1, CE2, CZ, OH, H, HA, HB2, HB3, HD1, HD2, HE1, HE2, HH
Ala	N, CA, C, O, CB, H, HA, HB1, HB2, HB3
Phe	N, CA, C, O, CB, CG, CD1, CD2, CE1, CE2, CZ, H, HA, HB2, HB3, HD1, HD2, HE1, HE2, HZ
Gly	N, CA, C, O, H, HA2, HA3
Ile	N, CA, C, O, CB, CG1, CG2, CD1, H, HA, HB, HG12, HG13, HG21, HG22, HG23, HD11, HD12, HD13
Leu	N, CA, C, O, CB, CG, CD1, CD2, H, HA, HB2, HB3, HG, HD11, HD12, HD13, HD21, HD22, HD23
Trp	N, CA, C, O, CB, CG, CD1, CD2, NE1, CE2, CE3, CZ2, CZ3, CH2, H, HA, HB2, HB3, HD1, HE1, HE3, HZ2, HZ3, HH2
Met	N, CA, C, O, CB, CG, SD, CE, H1, H2, H3, HA, HB2, HB3, HG2, HG3, HE1, HE2, HE3
Pro	N, CA, C, O, CB, CG, CD, HA, HB2, HB3, HG2, HG3, HD2, HD3
Val	N, CA, C, O, CB, CG1, CG2, H, HA, HB, HG11, HG12, HG13, HG21, HG22, HG23
